# Improved empirical antibiotic treatment of sepsis after an educational intervention: the ABISS-Edusepsis study

**DOI:** 10.1186/s13054-018-2091-0

**Published:** 2018-06-22

**Authors:** Ricard Ferrer, María Luisa Martínez, Gemma Gomà, David Suárez, Luis Álvarez-Rocha, María Victoria de la Torre, Gumersindo González, Rafael Zaragoza, Marcio Borges, Jesús Blanco, Eduardo Palencia Herrejón, Antonio Artigas, for the ABISS-Edusepsis Study group, Gemma Gomà, María Luisa Martínez, Antonio Artigas, María del Mar Cruz, Sandra Barbadillo, Francisco Fernández, Alberto Pensado Castiñeiras, Ma Teresa Rey Rilo, Luis Alvarez Rocha, Belén Jiménez Bartolomé, Juan Diego Jiménez Delgado, Demetrio Carriedo Ule, Ana María Domínguez Berro, Francisco Javier Díaz Domínguez, Juan Machado Casas, Clara Laplaza Santos, Manuel García-Montesinos, Enrique Maraví Poma, Víctor López Ciudad, Pablo Vidal Cortés, Miguel Martínez Barrio, Ma Jesús López Pueyo, María Jesús López Cambra, Pau Torrabadella, Álvaro Salcedo, Claudio Durán, Iratxe Seijas, Teresa Recio Gómez, Ángel Arenzana, Izaskun Azkarate, Sandra Rodríguez Bolaño, Pablo Olivares García, Jordi Solé Violán, Gerardo Aguilar Aguilar, Ángel Rodríguez Rencinas, Marta Paz Pérez, Elena Pérez Losada, Fernando Martínez Sagasti, José Luis García Allut, Fernando Díez Gutiérrez, Francisco Gandía, Amanda Francisco Amador, Ramón Vegas Pinto, Pilar Martínez Trivez, Nieves García Vázquez, Luis Zapata, Paula Vera, Eduardo Antón, Juan Carlos Yébenes, María de las Olas Cerezo Arias, Francisco García delgado, Javier Fierro Rosón, Josefa Peinado Rodríguez, María Álvarez, Paco Álvarez Lerma, Francisco Valenzuela, Patricia Albert de la Cruz, Rafael Blancas Gómez-Casero, Montserrat Sisón Heredia, Perico Olaechea, Celia Sañudo, José Manuel Gutiérrez Rubio, Roberto Reig Valero, Hasania Abdel-Hadi Álvarez, Leandro Fajardo Feo, Pau Garro, Francisco Navarro Pellejero, Ana Esther Trujillo Alonso, Rosa Catalán, Assumpta Rovira, Nicolás Rico, José Manuel Allegue Gallego, José Córdoba Alonso, Dolores Ocaña, Juan Mora Ordóñez, Manuel Salido Mota, Ma José Tolón Herrera, Paloma Dorado, Arantxa Lander Azcona, Diego Mendoza, Francisca Prieto, Ma Carmen Ramagge Martín, José Ignacio Ayestarán Rota, Marcio Borges, Enrique Piacentini, Ricard Ferrer, Josep Maria Sirvent, Cristina Murcia, Gina Rognoni, José Antonio Gonzalo, Diego Parra Ruiz, Natalia Bretón Díez, José Ignacio Argüelles Antuña, Leonardo Lorente Ramos, Helena Yáñez, Ana Loza, Borja Suberbiola, Domingo Ruiz de la Cuesta Martín, María del Mar Martín Velasco, Antonio Pontes Moreno, Rafael León López, Juan Carlos Pozo, Luis Tamayo Lomas, Jesús Blanco, Arturo Muriel, José Ángel Berezo, Paula Ramírez, Miguel Ángel Chiveli Monleón, Juan Carlos Ruiz Rodríguez, Jesús Caballero, Adolf Ruiz, Alejandra García, Jordi Riera, Javier Sarrapio, Francesc Sanpedro, José Carlos Martin, Tatiana Acero, Ana Carolina Caballero, Silvia María Cortés Díaz, M. Victoria de la Torre, Begoña Mora Ordóñez, José Garnacho Montero, Eduardo Palencia Herrejón, Begoña Bueno García, Gumersindo González-Díaz, Andrés Carrillo, Pedro Jesús Domínguez García, Ruth Jorge García, Almudena Simón, José Carlos Torralba Allué, Teresa Recio Gómez, Ricardo Díaz Abad, Mar Gobernado, Francisco Guerrero Gómez, José Castaño Pérez, Fernando Bueno Andrés, Elena Bustamante Munguira, Gaspar Tuero, José Francisco Olea Parejo, Miguel Soto, Susana Sancho Chinesta, Rafa Zaragoza, Carmen Fernández González, Manuel Castellano, José María Bonell, Ma Jesús Broch Porcar, Néstor Bacelar, Isabel Cremades, Miguel Valdivia, Pedro Galdós

**Affiliations:** 1 Intensive Care Department, Shock, Organ Dysfunction and Resuscitation Research Group, Vall d’Hebron Research Institute, Vall d’Hebron University Hospital, Autonomous University of Barcelona, Barcelona, Spain; 2 CIBER Enfermedades Respiratorias, Madrid, Spain; 3 Intensive Care Department, Hospital Universitario General de Catalunya, Autonomous University of Barcelona, Sant Cugat del Vallés, Spain; 4 Intensive Care Department, Corporación Sanitaria Universitaria Parc Taulí, Autonomous University of Barcelona, Sabadell, Spain; 5 Epidemiology and Assessment Unit, Fundació Parc Taulí, Autonomous University of Barcelona, Sabadell, Spain; 6 Intensive Care Department, Hospital Universitario de la Coruña, A Coruña, Spain; 7 Intensive Care Department, Hospital Universitario Virgen de la Victoria, Málaga, Spain; 8 Intensive Care Department, Hospital General Universitario Morales Meseguer, Murcia, Spain; 9 Intensive Care Department, Hospital Universitario Doctor Peset, Valencia, Spain; 10 Intensive Care Department, Hospital Son Llatzer, Palma de Mallorca, Spain; 11 Intensive Care Department, Hospital Universitario Rio Hortega, Valladolid, Spain; 12 Intensive Care department, Hospital Universitario “Infanta Leonor”, Madrid, Spain

**Keywords:** Sepsis, Septic shock, Quality improvement, Timing of antibiotics, De-escalation, Hospital mortality

## Abstract

**Background:**

Early appropriate antibiotic treatment is essential in sepsis. We aimed to evaluate the impact of a multifaceted educational intervention to improve antibiotic treatment. We hypothesized that the intervention would hasten and improve the appropriateness of empirical antibiotic administration, favor de-escalation, and decrease mortality.

**Methods:**

We prospectively studied all consecutive patients with sepsis/septic shock admitted to 72 intensive care units (ICUs) throughout Spain in two 4-month periods (before and immediately after the 3-month intervention). We compared process-of-care variables (resuscitation bundle and time-to-initiation, appropriateness, and de-escalation of empirical antibiotic treatment) and outcome variables between the two cohorts. The primary outcome was hospital mortality. We analyzed the intervention’s long-term impact in a subset of 50 ICUs.

**Results:**

We included 2628 patients (age 64.1 ± 15.2 years; men 64.0%; Acute Physiology and Chronic Health Evaluation (APACHE) II, 22.0 ± 8.1): 1352 in the preintervention cohort and 1276 in the postintervention cohort. In the postintervention cohort, the mean (SD) time from sepsis onset to empirical antibiotic therapy was lower (2.0 (2.7) vs. 2.5 (3.6) h; *p* = 0.002), the proportion of inappropriate empirical treatments was lower (6.5% vs. 8.9%; *p* = 0.024), and the proportion of patients in whom antibiotic treatment was de-escalated was higher (20.1% vs. 16.3%; *p* = 0.004); the expected reduction in mortality did not reach statistical significance (29.4% in the postintervention cohort vs. 30.5% in the preintervention cohort; *p* = 0.544). Gains observed after the intervention were maintained in the long-term follow-up period.

**Conclusions:**

Despite advances in sepsis treatment, educational interventions can still improve the delivery of care; further improvements might also improve outcomes.

**Electronic supplementary material:**

The online version of this article (10.1186/s13054-018-2091-0) contains supplementary material, which is available to authorized users.

## Background

Sepsis, a life-threatening organ dysfunction due to dysregulated host response to infection, is common and its incidence seems to be increasing [1, 2]. About a quarter of patients with sepsis go on to develop septic shock [3, 4]. Sepsis is associated with high morbidity and mortality [1, 2, 5]. The Surviving Sepsis Campaign (SSC), an international effort to optimize treatment for sepsis through evidence-based guidelines, has resulted in sustained continuous quality improvement associated with decreased mortality [6]. In Spain, a nationwide effort based on the SSC, the EDUSEPSIS intervention, succeeded in improving process-of-care and outcome measures [7].

Infection control is the cornerstone of treatment, and both the timeliness and appropriateness of empirical antibiotic treatment are considered essential aspects of sepsis management [8, 9]. The SSC guidelines recommend administering empirical broad-spectrum antibiotics within 1 h of identification of sepsis [10]. This recommendation is supported by a secondary analysis of the Edusepsis study, which showed that early administration of broad-spectrum empirical antibiotics was the component of the SSC bundles most strongly associated with increased survival [11]. Several studies have identified worse outcomes associated with delays in administering appropriate antibiotic treatment [1, 2, 12–15] and with inappropriate antibiotic therapy [12, 13, 16]. The SSC guidelines also recommend reassessing antibiotic treatment to determine whether de-escalation is possible, and failure to de-escalate might be associated with worse outcomes [8, 16, 17] and could lead to development of resistant microbes [8, 18].

Given the importance of infection control in sepsis management, we designed a multifaceted educational intervention to improve antimicrobial therapy in patients with sepsis: the Antibiotic Intervention in Severe Sepsis (ABISS) study. We hypothesized that the intervention would decrease the time to the administration of empirical antibiotics, increase the proportion of patients receiving appropriate empirical antibiotics, favor de-escalation, and decrease mortality.

## Methods

Through the Edusepsis study group and the Spanish Society of Critical Care Medicine’s working group on infectious disease and sepsis, we invited 115 centers throughout Spain to participate in a national educational intervention to improve infection control: the ABISS-Edusepsis study. A total of 72 medical-surgical intensive care units (ICUs) located throughout Spain took part. The study was approved by our institutional review board (reference 2,011,521) and the ethics committees at each participating center approved the study protocol and waived the need for informed consent because the intervention was a quality improvement program and patients’ anonymity was guaranteed.

### Study design

We designed a before-and-after study to compare a preintervention cohort consisting of all consecutive patients with severe sepsis or septic shock admitted to the participating ICUs in the 4-month period before the educational program began (April–July 2011) against a postintervention cohort in the 4-month period immediately after the intervention (April–July 2012). The intervention took place over a 3-month period (January–March 2012) during which no patient data were collected. Furthermore, to assess the long-term impact of the intervention, we analyzed a third cohort 6 months after the postintervention period (January–April 2013).

The study sites, study design, data collection, and quality-control measures are detailed in Additional file 1. Severe sepsis was defined as sepsis associated with organ dysfunction unexplained by other causes. Septic shock was defined as sepsis associated with systolic blood pressure < 90 mmHg, mean arterial pressure < 65 mmHg, or a reduction in systolic blood pressure > 40 mmHg from baseline despite adequate volume resuscitation [19]. The onset of sepsis (T_0_) was determined according to the patient’s location within the hospital when sepsis was diagnosed: we used the time of triage for patients diagnosed in the emergency department and searched the clinical documentation for clues indicating the time of diagnosis for patients diagnosed in the wards or the ICU (see Additional file 1).

### Intervention

Between January and March 2012, we implemented a multifaceted educational program to train physicians and nursing staff in the emergency department, medical and surgical wards, and ICU in sepsis care, with special emphasis on antimicrobial management. Training included primary sepsis care focused on timing and strategy of empiric measures against infection. The intervention consisted of educational outreach, periodic reminders, auditing and feedback, and a videogame. The educational outreach included interactive educational sessions in which the local leader gave a 30-min slide presentation based on the SSC guidelines recommendations focused on the importance of (a) infection control in sepsis, (b) the timeliness and appropriateness of empirical antibiotic administration, and (c) de-escalation of antibiotic treatment. Each center was provided with pocket guides and posters with recommendations from the Spanish Society of Critical Care Medicine and Coronary Units. To facilitate antibiotic prescription, researchers preferentially used their local guideline or an electronic clinical decision support system (www.es.dgai-abx.de). Attendees received weekly email and cellphone text reminders reiterating the most important points from the educational outreach program. Additionally, a videogame was developed to provide staff attending septic patients (http://www.edusepsis.org/en/training.html) with opportunities to put the guidelines into practice with simulations. The educational intervention is detailed in Additional file 2.

### Process-of-care and outcome measurements

We recorded demographic characteristics (age and sex), Charlson Comorbidity Score, diagnosis on admission (medical, emergency surgery, elective surgery), source of sepsis, type of infection (community-acquired, healthcare-related, hospital-acquired, or ICU-acquired), Sequential Organ Failure Assessment (SOFA) scale on admission, and worst Acute Physiology and Chronic Health Evaluation (APACHE) II score during the first 24 h in the ICU.

We recorded the following process-of-care variables related to empirical antibiotic treatment: drugs administered, time initiated, appropriateness, and de-escalation. Time to first antibiotic administration is reported as the difference between onset of sepsis and first antibiotic administration. Empirical treatment was classified as appropriate (when ≥ 1 of the drugs administered was considered effective based on the susceptibility in the antibiogram of the causative microorganism isolated in cultures), inappropriate (when the above criterion was not met), or indeterminate (when no causative microorganism was isolated in cultures or no cultures were taken). Antibiotic strategies once culture results were available were classified as “de-escalation” (switch to or interruption of a drug class resulting in a less broad spectrum of coverage, “no change” (empirical therapy maintained without modification), or “change” (due to poor clinical evolution, uncovered microorganism, possible toxicity, or other reasons).

We also recorded time from T_0_ to other acts and targets prescribed in the SSC guidelines: measuring serum lactate, obtaining blood cultures, and administering fluids and/or vasopressors in patients with hypotension and/or lactate > 4 mmol/L [10].

Patients were followed up until death or hospital discharge. The primary outcome variable was hospital mortality. Secondary outcome measures included days on mechanical ventilation, days on vasopressors, hospital and ICU lengths of stay, and ICU mortality.

### Statistical analysis

Descriptive statistics included frequencies and percentages for categorical variables and means and standard deviations (SD) for continuous variables. To compare categorical variables, we used the chi-squared (χ2) test or Fisher’s exact test as appropriate. To compare continuous variables, we used Student’s *t* test or the Mann-Whitney U test as appropriate.

To assess the effectiveness of the intervention, we compared the values of the process and outcome variables recorded in the preintervention cohort against those recorded in the postintervention cohort. To assess long-term effectiveness, we compared the values recorded in the long-term follow-up period against those recorded in the postintervention period in the same subset of hospitals.

We used multivariate linear regression to determine the association between the intervention and time to antibiotics after adjusting for possible confounders. Moreover, as a sensitivity analysis, we performed segmented regression analysis to estimate the size of the effect of the intervention for reducing time to antibiotic.

We used multivariate stepwise logistic regression to assess the impact of the intervention on outcome (hospital mortality). Variables entered in the logistic regression model were those with a relationship in the univariate analysis (*p* ≤ 0.1) or with a potential plausible relationship with the outcome. The final model included the intervention, age, sex, comorbidities, APACHE II, SOFA, type of infection, and source of sepsis as independent variables. Statistical tests were two-tailed. We used SPSS version 15.0 (SPSS, Chicago, IL, USA) for all analyses.

## Results

### Patients characteristics

A total of 2628 patients (mean age 64.1 (15.2) years; mean APACHE II score 22.0 (8.1); 64% male) were included during the preintervention (*n* = 1352) and postintervention periods (*n* = 1276). Table 1 reports the demographic and clinical characteristics of the patients in these two cohorts. Compared to patients in the preintervention cohort, those in the postintervention cohort had slightly less severe illness and had a greater proportion of community-acquired infections. There were no differences in age, sex, or SOFA scores. Diagnosis on admission was mainly medically or surgically urgent in both cohorts and the main sources of sepsis in both periods were pneumonia and acute abdominal infections.

**Table 1:** Demographic and clinical characteristics of patients

Patient Characteristic	Preintervention cohort (*n* = 1352)	Postintervention cohort (*n* = 1276)	*P*
General data			
Age (years), mean (SD)	64.3 (15.3)	63.8 (15.1)	0.400
Sex (male), *n* (%)	858 (63.5)	824 (64.6)	0.552
APACHE II, mean (SD)	22.5 (8.1)	21.4 (8.0)	0.001
SOFA, mean (SD)	8.7 (3.5)	8.5 (3.5)	0.102
Charlson, mean (SD)	2.7 (2.3)	2.7 (2.3)	0.391
Source of sepsis, *n* (%) 0.018			
Pneumonia	454 (33.6)	403 (31.6)	
Acute abdominal infection	452 (33.4)	431 (33.8)	
Urinary tract infection	229 (16.9)	209 (16.4)	
Soft-tissue infection	82 (6.1)	98 (7.7)	
Meningitis	26 (1.9)	43 (3.4)	
Catheter-related bacteremia	30 (2.2)	19 (1.5)	
Other infections	79 (5.8)	73 (5.7)	
Type of infection, *n* (%) 0.013			
Community	802 (59.3)	835 (65.4)	
Nosocomial	302 (22.3)	249 (19.5)	
ICU	65 (4.8)	51 (4)	
Healthcare-related	183 (13.5)	141 (11.1)	
Diagnosis on admission, *n* (%) 0.817			
Medical	950 (70.3)	891 (69.8)	
Urgent surgical	318 (23.5)	311 (24.4)	
Non-urgent surgical	84 (6.2)	74 (5.8)	

*Abbreviations*: *APACHE II* Acute Physiology and Chronic Health Evaluation II, *SOFA* Sequential Organ Failure Assessment, *ICU* intensive care unit, *SD* standard deviations

**Fig. 1:**
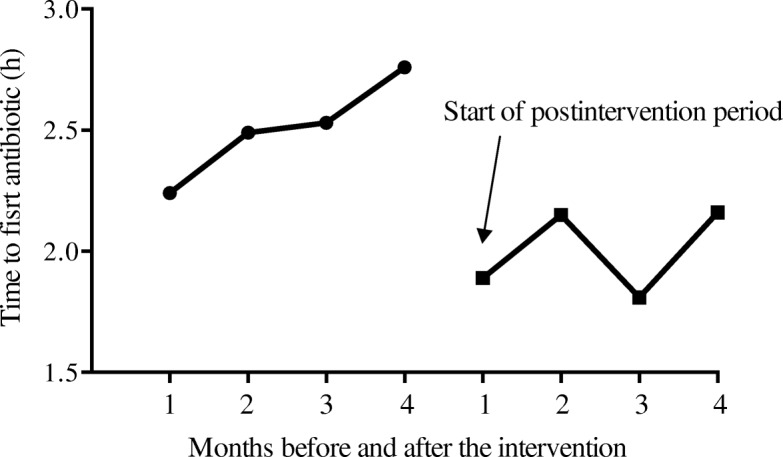
Mean time to first antibiotic after sepsis onset before and after the educational intervention, excluding patients with previous antibiotic treatment (*n* = 858)

Compliance with three of the six tasks in the 6-h resuscitation bundle (lactate measurement, blood cultures before antibiotics, and early administration of broad spectrum antibiotics) improved significantly after the intervention (see Fig. 1). Compared to the preintervention cohort, the mean time from sepsis onset to empirical antibiotic therapy was shorter in the postintervention cohort (2.0 (2.7) vs. 2.5 (3.6) hours; *p* = 0.002). The proportion of patients receiving inappropriate empirical antibiotic treatment decreased from 8.9% in the preintervention cohort to 6.5% in the postintervention cohort (*p* = 0.024) and the proportion of patients in whom antibiotic treatment was de-escalated was higher in the postintervention cohort (20.1% vs. 16.3% in the preintervention cohort; *p* = 0.004) (see Fig. 1).

After adjustment for severity of illness, type and source of infection, and demographic characteristics, mean time to first antibiotic after sepsis onset was significantly lower in the postintervention group (− 0.45 (95% CI − 0.75 to − 1.56); *p* = 0.003) (see Additional file 3: Table S1).

**Fig. 2:**
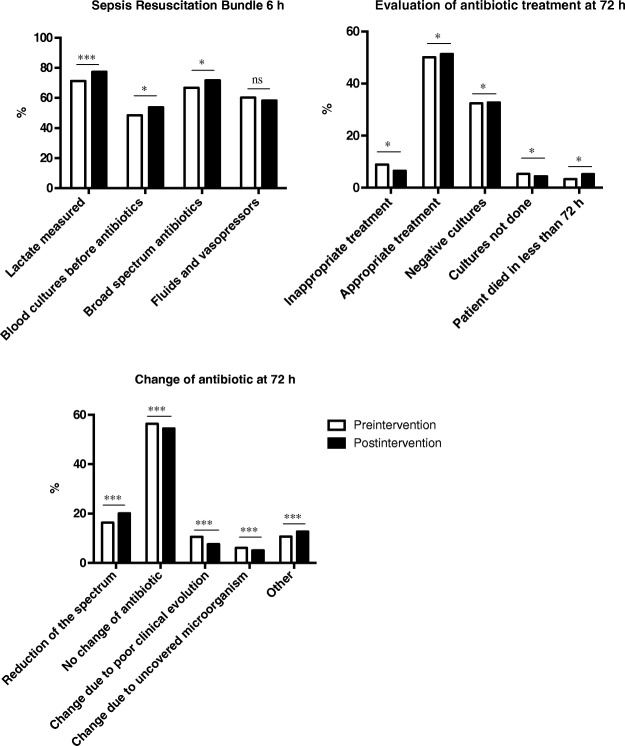
Compliance with process-of-care measures in the preintervention vs. postintervention cohort. **a** The proportion of adherence to the resuscitation bundle in the preintervention vs. postintervention cohort. **b** Evaluation of treatment at 72 h. **c** Change of antibiotic at 72 h. NS, not statistically significant; *p < 0.05; ***p < 0.005

Figure 2 shows the time series of mean time to first antibiotic after sepsis onset per month in the preintervention and postintervention periods. The segmented regression analysis found a significant change in level (− 0.92 (95% CI − 1.51 to − 0.33); *p* = 0.010), indicating an abrupt intervention effect (see Additional file 4: Table S2).

Table 2 reports the outcome variables. No significant differences were observed in any of the outcome variables. Overall hospital mortality (29.9%) did not differ between cohorts. Multivariable logistic regression (Table 3) to adjust for possible confounders showed no relationship between intervention and hospital mortality.

**Table 2:** Outcome measurements in the preintervention vs. postintervention cohort

Outcome measurements	Preintervention cohort (*n* = 1352)	Postintervention cohort (n = 1276)	*P*
Duration of MV, days mean (SD)	6.9 (14.4)	6.6 (12.4)	0.577
Duration of vasopressors, days mean (SD)	4.0 (8.0)	4.3 (7.0)	0.369
ICU stay, days mean (SD)	12.0 (17.0)	11.5 (14.9)	0.443
Hospital stay, days mean (SD)	30.0 (29.7)	28.4 (28.9)	0.161
Mortality, *n* (%)			
ICU	332 (24.6)	301 (23.6)	0.562
Hospital	412 (30.5)	375 (29.4)	0.544

*Abbreviations*: *ICU* intensive care unit, *MV* mechanical ventilation, *SD* standard deviations

**Table 3:** Multivariate analysis of risk factors for hospital mortality

Factors	OR	95% CI	*P*
Interventional cohort	1.08	0.89–1.31	0.419
Age^a^	1.02	1.01–1.03	< 0.001
Sex^b^	0.82	0.67–1.01	0.057
SOFA^a^	1.11	1.07–1.15	< 0.001
APACHE II^a^	1.08	1.06–1.10	< 0.001
Charlson^a^	1.06	1.02–1.11	0.005
Type of infection^c^			
Nosocomial	2.03	1.61–2.56	< 0.001
ICU	2.49	1.61–3.87	< 0.001
Healthcare-related	1.33	0.99–1.79	0.058
Source of sepsis^d^			
Acute abdominal infection	0.79	0.63–1.00	0.051
Urinary tract infection	0.25	0.18–0.35	< 0.001
Meningitis	1.11	0.60–2.05	0.742
Soft-tissue infection	0.69	0.47–1.03	0.072
Catheter-related bacteremia	0.54	0.27–1.09	0.084
Other infections	1.00	0.66–1.52	0.999

*Abbreviations*: *SOFA* Sequential Organ Failure Assessment, *APACHE II* Acute Physiology and Chronic Health Evaluation II, *ICU* intensive care unit

^a^Per each point of increase

^b^Compared with male sex

^c^Compared to community-acquired infection.

^d^Compared to pneumonia

### Long-term follow up

Fifty centers participated in a 4-month follow-up study to measure the long-term effects of the intervention (*n* = 830 patients). Compared to patients from the postintervention cohort, those in the long-term cohort had lower mean Charlson score and there was a higher proportion of patients with pneumonia (see Additional file 5: Table S3).

The percentage of patients in whom care complied with resuscitation measures was stable with respect to the postintervention cohort. Time from sepsis onset to empirical antibiotic therapy increased slightly but not significantly in the long-term cohort and the proportion of patients in whom antibiotic treatment was de-escalated remained unchanged (see Additional file 6: Table S4).

No significant differences were observed in the outcome variables (see Additional file 7: Table S5). Multivariable logistic regression showed no relationship between the intervention and hospital mortality (see Additional file 8: Table S6).

## Discussion

This large-scale multifaceted educational intervention improved the overall use of antibiotics in sepsis, improving efficacy by lowering the time from sepsis onset to antibiotic treatment and increasing the proportion of patients who received appropriate empirical treatment and also improving safety by increasing the proportion of patients who received appropriate de-escalation. Importantly, gains observed after the intervention were maintained in the long-term follow-up period.

These results lend strength to a growing body of literature showing that educational interventions can improve the process of care in different contexts and conditions [15, 20–24]. However, Ramsay et al. [25] reviewed the effectiveness of interventions to improve antibiotic prescribing in hospital inpatients and concluded that most studies supporting these interventions had fundamental flaws in design and/or execution, pointing out that segmented regression analyses are recommendable when analyzing the effects of interventions on process measures. In our data, these analyses estimating intervention effects in interrupted time series studies showed a significant change in level, indicating an abrupt intervention effect, and thus confirming the results of our multivariate linear regression and strengthening the conclusion that the intervention reduced the time to first antibiotic.

Since multifaceted interventions appear to be more effective than more limited approaches to changing behavior [26, 27], we aimed to strengthen the intervention by including various approaches to transferring and reinforcing knowledge. Two of the approaches we used, educational outreach and auditing and feedback, are well-established approaches to knowledge translation; the other two, weekly reminders and an educational game to increase awareness and improve adherence to guidelines, are supported by more limited experiences [28–31].

Despite significant improvement in antibiotic treatment, no significant decrease in mortality was observed. One factor that probably contributed to our not identifying an impact on mortality is that our study focused mainly on improving antimicrobial treatment. It is unlikely that a limited intervention at a single point in time would have a profound impact on survival. Seymour et al. [32] in a study with more than 49,000 emergency department patients with sepsis showed a linear association between time to antibiotic and mortality. This study was done after the implementation of a statewide mandate requiring protocolized sepsis care rather than after educational intervention, and compliance with the 3-h bundle was very high.

The decrease in mortality associated with the reduction in time to antibiotic observed in the present study was comparable to forecasts based on previous research. In the Seymour et al. study [32], the odds of death increased by 4% for every 1-h delay in receiving antibiotics, and Kumar et al. [14] found mortality decreased 7% per hour of reduction in time to antibiotics. We observed a half-hour reduction in time to antibiotic. Interestingly, the observed mortality reduction was practically the same as in the Kumar et al. study: 7.2% per hour (3.6% per half-hour, from 30.5% mortality in the preintervention to 29.4% in the postintervention period).Unfortunately, however, our study was underpowered to detect this difference in mortality; to achieve a statistically significant result with a type I error rate of 5% and 80% power would have required the inclusion of 50,000 patients.

Although delaying antibiotic administration in patients with sepsis is inadvisable, the evidence supporting the mortality benefits of early antibiotic administration is inconclusive. Ferrer et al. [15], in a study with more than 17,000 patients, confirmed that delayed antibiotic administration is associated with increased hospital mortality. More recently, in a large multicenter sample of patients with sepsis, Liu et al. [33] found a linear association between delays in antibiotic administration and mortality; patients with septic shock received the greatest benefit from early administration. Whiles et al. [34] similarly reported an 8% increase in the chance of developing septic shock for each hour of delay in antibiotic administration. Therefore, the mortality benefits of early antibiotic administration are probably especially important in the most critically ill patients.

A recent meta-analysis of six studies including more than 16,000 patients found no significant mortality benefit of administering antibiotics within 3 h of emergency department triage or within 1 h of recognition of shock, although the inclusion of studies with small samples and heterogeneity among studies may limit the conclusions [35]. In a cluster randomized trial to evaluate the effect of a multifaceted educational intervention for anti-infectious measures on sepsis mortality, the MEDUSA study group [36] found no association between the intervention and impact on time to empiric antibiotic or mortality; however, the authors concluded the intervention was insufficient, inconsistent, and mainly applied only in the ICU. Our results are similar to those of the pediatric substudy of the ABISS-Edupsesis project [37], a multifaceted educational intervention in children with sepsis and septic shock, which found decreased time to antibiotic administration but not decreased mortality after the intervention, probably due to the small sample size.

Singer [38] questions the importance of earlier antibiotic treatment mainly because the risk of increasing antimicrobial resistance. Our intervention increased the proportion of patients receiving appropriate de-escalation from 16.3% to 20.1%, and these improvements were maintained in the long-term follow-up period. In a recent prospective study, about 35% of patients with sepsis received appropriate de-escalation and de-escalation was associated with lower mortality [39]. A multicenter non-blinded randomized non-inferiority trial in 1116 patients with sepsis found that de-escalation did not worsen patient outcomes [40].

Efforts to improve the treatment of sepsis, especially those targeting empirical antibiotic administration, need to encompass all levels of care. We targeted all professionals caring for septic patients. For knowledge transfer to benefit patients, it is often necessary to reorganize how care is delivered [41]. The key to improving outcomes in sepsis is motivating professionals to implement evidence-based measures and providing them with feedback about the impact of these measures [42]. To this end, it is important to monitor process-of-care variables and outcome variables. One of the greatest benefits of interventions like ours is their contribution to shaping a culture that fosters the desire to improve, and an ongoing commitment to excellence in patient care [41].

### Limitations

The before-and-after design of our study has inherent weaknesses. The influence of secular trends can be difficult to separate from the effects of the intervention in studies employing before-and-after designs. In these cases, experts recommend using a stepped-wedge design. However, no other major changes in protocol were instituted in the relatively short gaps between the three periods. Not using a control group makes it effectively impossible to ensure that the changes observed after the intervention would not have happened anyway. Although using centers where nothing was done to improve antibiotic therapy for sepsis as a control group might have enabled us to sort out the effects of a possible secular trend, we considered this approach might be unethical. Another argument against uncontrolled before-and-after studies is that it is impossible to ensure that the intervention site is representative and change is merely an expression of regression to the mean [25]; however, the large number of centers participating in our study safeguard against this. We also performed a segmented regression analysis, a powerful method for estimating intervention effects in interrupted time series [43].

Although our intervention employed a broad multifaceted approach, other measures such as real-time automated alerting to remind clinicians were not included, and this may be partly responsible for our failure to find a strong effect on outcome. The long-term follow-up analysis (6 months after the postintervention period) might not be late enough to assess the long-term impact of the intervention. Our earlier study found that some improvements were maintained after 1 year [7], and the very long-term impact of the interventions was recently confirmed in another study between 2005 and 2011 that identified dramatically decreased mortality related to severe sepsis/septic shock [44].

Despite these limitations, our study has noteworthy strengths. The large number of ICUs that participated enabled us to prospectively enroll and follow large numbers of patients with sepsis in each data collection period and increases the likelihood that our results can be applied in other contexts. Our strict quality control helped ensure a homogeneous database and the validity of our data.

## Conclusion

Sepsis is a time-dependent condition in which early empirical antibiotic treatment can improve survival. Both the time from onset to administration of antibiotics and antibiotic de-escalation are modifiable factors worthy of our attention. The ABISS intervention reduced the time to antibiotic administration and the proportion of patients in whom antibiotic treatment was de-escalated, thus demonstrating that despite advances in sepsis treatment in recent years, educational interventions can still improve the delivery of care. Further improvements might also improve outcomes.

## Additional files

Additional file 1: ** Appendix 1.** Appendix describing in detail the study design, the approach to data collection, and the quality-control measures to ensure data reliability. (DOC 27 kb)

Additional file 2: ** Appendix 2.** Appendix describing in detail the educational intervention. (DOC 25 kb)

Additional file 3: ** Table S1.** Multivariate linear regression for time to antibiotic. (DOC 38 kb)

Additional file 4: **Table S2.** Segmented regression model for time in hours to first antibiotic. (DOC 29 kb)

Additional file 5: **Table S3.** Demographic and clinical characteristics of patients in the long-term cohort. (DOC 48 kb)

Additional file 6: ** Table S4.** Compliance with process-of-care measurements in the long-term cohort. (DOC 44 kb)

Additional file 7: **Table S5.** Outcome measurements in the long-term cohort. (DOC 31 kb)

Additional file 8: **Table S6.** Multivariate analysis of factors associated with mortality in the long-term cohort. (DOC 37 kb)

## Abbreviations

ABISS: Antibiotic Intervention in Severe Sepsis
APACHE: Acute Physiology and Chronic Health Evaluation
CI: Confidence interval
ICU: Intensive care units
IQR: Interquartile range
SD: Standard deviation
SOFA: Sequential Organ Failure Assessment
SSC: Surviving Sepsis Campaign
T_0_: Onset of sepsis
