# Supplementary material for: Improved empirical antibiotic treatment of sepsis after an educational intervention: the ABISS-Edusepsis study
Source: Crit Care. 2018 Jun 22;22:167. doi: 10.1186/s13054-018-2091-0 (PMC6013897; doi:10.1186/s13054-018-2091-0)
Supplement: Supplementary file 1 — Appendix 1. Appendix describing in detail the study design, the approach to data collection, and the quality-control measures to ensure data reliability. (DOC 27 kb) [file 13054_2018_2091_MOESM1_ESM.doc]

**Additional file 1:** Appendix describing in detail the study design, the approach to data collection, and the quality-control measures to ensure data reliability.

**Study Sites**

The steering committee of the ABISS-Edusepsis study defined the project’s purpose, timeline, interventions, and design. Through the Spanish Society of Intensive Care, all Spanish ICUs for adults (115 units) were invited to participate. No fees were provided for participation. ICUs were not asked to provide reasons for not participating. Seventy-two medical-surgical Spanish ICUs homogeneously distributed around the country were included in the study. All ICUs were closed units with a critical care specialist on hand 24 hours per day, 365 days per year. The general coordinating center was located at the Critical Care Center of the Hospital Universitario Mútua de Terrassa, Barcelona. Each ICU belonged to a geographical area coordinated by an area coordinator and at least 1 physician was designated principal investigator in each center.

**Patients**

All ICU admissions from the emergency department or medical or surgical wards and all ICU patients were actively screened daily for severe sepsis or septic shock. Patients who received initial infection control measures for sepsis in another hospital were excluded.

Severe sepsis was defined as sepsis associated with at least one acute organ dysfunction: (1) respiratory dysfunction (bilateral pulmonary infiltrates with PaO2/FIO2 ratio < 300 mmHg); (2) renal dysfunction (urine output < 0.5 mL/kg per hour for ≥2 hours or creatinine increase > 0.5 mg/dL or creatinine level > 2.0 mg/dL); (3) coagulation abnormalities (international normalized ratio >1.5 or partial thromboplastin time > 60 seconds); (4) thrombocytopenia (platelets <100 x 10^3^/μL); (5) hyperbilirubinemia (total plasma bilirubin >2.0 mg/dL); (6) hypoperfusion (lactate >3 mmol/l); or (7) hypotension (systolic blood pressure < 90 mmHg, mean arterial pressure < 65 mm Hg, or a reduction in systolic blood pressure > 40 mm Hg from baseline measurements). Septic shock was defined as sustained acute circulatory failure (systolic blood pressure < 90 mm Hg, mean arterial pressure < 65 mm Hg, or a reduction in systolic blood pressure < 40 mm Hg from baseline) despite adequate volume resuscitation (19).

The onset of sepsis (T_0_) was determined according to the patient’s location within the hospital when sepsis was diagnosed. In patients diagnosed with sepsis in the emergency department, T_0_ was defined as the time of triage. For patients admitted to the ICU from the medical or surgical wards or other non-emergency department units, T_0_ was determined by searching the clinical documentation for the time of diagnosis of severe sepsis. This might include, for example, a physician’s note or timed and dated orders, a timed and dated note of a nurse’s discussion of severe sepsis with a physician, or timed records initiating referral to the ICU for severe sepsis. If no time and date could be found by searching the chart, the default time of presentation was the time of admission to the ICU. Lastly, for patients who developed sepsis after admission to the ICU, the time of presentation was again determined on the basis of the clinical documentation.

**Data Collection and Quality Control**

Data were collected prospectively daily using an electronic database (EDICS, [www.edics.org](http://www.edics.org/)). Before data collection started, all investigators received detailed information explaining the aim of the study, instructions for data collection, and definitions for various items. The database included automated filters to check for incorrect entries and alert researchers to possible errors. For quality assurance purposes, two research nurses and a physician with experience in sepsis trials checked data for completeness, accuracy, and uniformity. Errors or blank fields generated queries that were returned to each center for correction.
