# Supplementary material for: Improved empirical antibiotic treatment of sepsis after an educational intervention: the ABISS-Edusepsis study
Source: Crit Care. 2018 Jun 22;22:167. doi: 10.1186/s13054-018-2091-0 (PMC6013897; doi:10.1186/s13054-018-2091-0)
Supplement: Supplementary file 2 — Appendix 2. Appendix describing in detail the educational intervention. (DOC 25 kb) [file 13054_2018_2091_MOESM2_ESM.doc]

**Additional file 2:** Appendix describing in detail the educational intervention.

To standardize the educational program, the general coordinating center organized meetings with the area coordinators and the principal investigator of the participating centers before and after each study period. Before the implementation of the educational program, the importance of the study was explained to the hospital manager at each participating center to ensure full institutional support. The general coordinating center provided specific material for this meeting with information regarding the epidemiology, morbidity, mortality, and costs of sepsis.

The principal investigator acted as local champion, charged with creation of a local multidisciplinary team with representatives of all pertinent stakeholders, including physicians and nurses from the ICU and infectious diseases, emergency, and internal medicine departments.

Between January and March 2012, the local multidisciplinary team at each hospital implemented a homogeneous predefined multifaceted educational program based on the SSC guidelines with special emphasis on antimicrobial management. The educational program was implemented in the emergency department, medical and surgical wards, and the ICU. The intervention consisted of educational outreach, periodic reminders, auditing and feedback, and a videogame. The educational outreach included interactive educational sessions in which the local leader gave a 30-minute slide presentation based on the SSC guidelines recommendations focused on the importance of infection control in sepsis. Each center was provided with pocket guides and posters with recommendations from the Spanish Society of Critical Care Medicine and Coronary Units. Posters were displayed in prominent places in the emergency departments, medical and surgical wards, and ICUs. Pocket versions were distributed to all participants in the educational program sessions. To facilitate antibiotic prescription, researchers used preferentially their local guideline or an electronic clinical decision support system ([www.es.dgai-abx.de](http://www.es.dgai-abx.de/)). Attendees received weekly email and cellphone text reminders reiterating the most important points from the educational outreach program. Additionally, a videogame was developed to provide staff attending septic patients with an opportunity to practice applying the guidelines in a simulated environment and to receive feedback about their performance (http://www.edusepsis.org/en/training.html).

Each center’s performance was audited and compared with the performance of the overall group, and local leaders received weekly feedback about their center’s performance and distributed these results to all staff. The general coordinating center maintained continuous contact with the principal investigator at each center through a mailing list. Moreover, after the educational program, a survey was distributed to all principal investigators to check that all participating centers had completed the educational program, to know the number and duration of lectures, and to know the principal investigator’s subjective evaluation of the main endpoints of the educational program, which were institutional support, creation of a multidisciplinary team, improvement in knowledge, and improvement in hospital processes.
