# Supplementary material for: Improved empirical antibiotic treatment of sepsis after an educational intervention: the ABISS-Edusepsis study
Source: Crit Care. 2018 Jun 22;22:167. doi: 10.1186/s13054-018-2091-0 (PMC6013897; doi:10.1186/s13054-018-2091-0)
Supplement: Supplementary file 3 — Table S1. Multivariate linear regression for time to antibiotic. (DOC 38 kb) [file 13054_2018_2091_MOESM3_ESM.doc]

**Additional file 3: Table 1. Multivariate linear regression for time to antibiotic**

| **Factors** | **Coefficient** | **95% CI** | **p** |
| --- | --- | --- | --- |
| **Post-intervention cohort^a^** | -0.45 | -0.75 to -1.56 | 0.003 |
| **Age^b^** | 0.01 | -0.006 to 0.02 | 0.380 |
| **Sex^c^** | -0.06 | -0.38 to -0.25 | 0.694 |
| **SOFA^b^** | 0.02 | -0.04 to 0.08 | 0.427 |
| **APACHE II^b^** | -0.01 | 0.03 to 0.22 | 0.739 |
| **CHARLSON^b^** | -0.23 | -0.09 to 0.04 | 0.466 |
| **Type of infection^d^** | | | |
| Nosocomial | 0.92 | 0.47 to 1.37 | <0.001 |
| ICU | 3.40 | 2.57 to 4.23 | <0.001 |
| Healthcare related | -0.19 | -0.27 to 0.66 | 0.415 |
| **Source of sepsis^e^** | | | |
| Acute abdominal infection | 0.31 | -0.07 to 0.69 | 0.11 |
| Urinary tract infection | -0.23 | -0.68 to 0.21 | 0.303 |
| Meningitis | 0.09 | -0.80 to 0.98 | 0.841 |
| Soft-tissue infection | 0.03 | -0.59 to 0.65 | 0.926 |
| Catheter-related bacteremia | -0.81 | -2.01 to 0.39 | 0.185 |
| Other infections | -0.02 | -0.75 to 0.71 | 0.954 |

Excluding patients with previous antibiotics (n = 858).

Abbreviations: SOFA, Sequential Organ Failure Assessment; APACHE II, Acute Physiology and Chronic Health Evaluation II; ICU, Intensive Care Unit.

^a^Compared with pre-intervention cohort.

^b^Per each point of increase

^c^Compared with female sex.

^d^Compared to community-acquired infection.

^e^Compared to pneumonia
