# Supplementary material for: Improved empirical antibiotic treatment of sepsis after an educational intervention: the ABISS-Edusepsis study
Source: Crit Care. 2018 Jun 22;22:167. doi: 10.1186/s13054-018-2091-0 (PMC6013897; doi:10.1186/s13054-018-2091-0)
Supplement: Supplementary file 4 — Table S2. Segmented regression model for time in hours to first antibiotic. (DOC 29 kb) [file 13054_2018_2091_MOESM4_ESM.doc]

**Additional file 4: Table 2.** Segmented regression model for time to first antibiotic in hours ^a^

|  | **Coefficient** | **95% CI** | **p** |
| --- | --- | --- | --- |
| **Intercept** | 2.25 | 1.87-2.63 | < 0.001 |
| **Change in level**  **(Post vs. pre-intervention)** | -0.92 | -1.51 - -0.33 | 0.010 |
| **Trend** | 0.11 | -0.03-0.23 | 0.092 |

^a^Excluding patients with previous antibiotics (n = 858).

Interaction between trend and intervention was not significant (p=0.288) and therefore was not included in the final model.
