# Supplementary material for: Improved empirical antibiotic treatment of sepsis after an educational intervention: the ABISS-Edusepsis study
Source: Crit Care. 2018 Jun 22;22:167. doi: 10.1186/s13054-018-2091-0 (PMC6013897; doi:10.1186/s13054-018-2091-0)
Supplement: Supplementary file 5 — Table S3. Demographic and clinical characteristics of patients in the long-term cohort. (DOC 48 kb) [file 13054_2018_2091_MOESM5_ESM.doc]

**Additional file 5: Table 3**. Demographic and clinical characteristics of patients in the long-term cohort

| **Patient Characteristic** | **Preintervention Cohort**  **(n= 1352)** | **Postintervention Cohort**  **(n= 1276)** | **Long-term Cohort**  **(n= 830)** | **p** |
| --- | --- | --- | --- | --- |
| **General data** | | | | |
| Age (years), mean (SD) | 64.3 (15.3) | 63.8 (15.1) | 63.5 (15.1) | 0.689 |
| Sex (male), n (%) | 858 (63.5) | 824 (64.6) | 513 (61.8) | 0.197 |
| APACHE-II, mean (SD) | 22.5 (8.1) | 21.4 (8.0) | 21.3 (7.8) | 0.591 |
| SOFA, mean (SD) | 8.7 (3.5) | 8.5 (3.5) | 8.7 (3.3) | 0.180 |
| Charlson, mean (SD) | 2.7 (2.3) | 2.7 (2.3) | 2.4 (2.2) | 0.007 |
| **Source of sepsis, n (%)** 0.004 | | | | |
| Pneumonia | 454 (33.6) | 403 (31.6) | 320 (38.6) |  |
| Acute abdominal infection | 452 (33.4) | 431 (33.8) | 270 (32.5) |  |
| Urinary tract infection | 229 (16.9) | 209 (16.4) | 131 (15.8) |  |
| Soft-tissue infection | 82 (6.1) | 98 (7.7) | 18 (2.2) |  |
| Meningitis | 26 (1.9) | 43 (3.4) | 51 (6.1) |  |
| Catheter-related bacteremia | 30 (2.2) | 19 (1.5) | 14 (1.7) |  |
| Other infections | 79 (5.8) | 73 (5.7) | 26 (3.1) |  |
| **Type of infection, n (%)** 0.317 | | | | |
| Community | 802 (59.3) | 835 (65.4) | 521 (62.8) |  |
| Nosocomial | 302 (22.3) | 249 (19.5) | 190 (22.9) |  |
| ICU | 65 (4.8) | 51 (4) | 33 (4) |  |
| Health care related | 183 (13.5) | 141 (11.1) | 86 (10.4) |  |
| **Diagnosis on admission, n (%)** 0.061 | | | | |
| Medical | 950 (70.3) | 891 (69.8) | 604 (72.8) |  |
| Urgent surgical | 318 (23.5) | 311 (24.4) | 196 (23.6) |  |
| Non-urgent surgical | 84 (6.2) | 74 (5.8) | 30 (3.6) |  |

Abbreviations: APACHE II, Acute Physiology and Chronic Health Evaluation II; SOFA, Sequential Organ Failure Assessment; ICU, intensive care unit.
