# Supplementary material for: Improved empirical antibiotic treatment of sepsis after an educational intervention: the ABISS-Edusepsis study
Source: Crit Care. 2018 Jun 22;22:167. doi: 10.1186/s13054-018-2091-0 (PMC6013897; doi:10.1186/s13054-018-2091-0)
Supplement: Supplementary file 6 — Table S4. Compliance with process-of-care measurements in the long-term cohort. (DOC 44 kb) [file 13054_2018_2091_MOESM6_ESM.doc]

**Additional file 6: Table 4.** Compliance with process-of-care measurements in the long-term cohort

| **Process-of-care** | **Preintervention Cohort**  **(n= 1352)** | **Postintervention Cohort**  **(n= 1276)** | **Long-term cohort**  **(n= 830)** | **p** |  | | |
| --- | --- | --- | --- | --- | --- | --- | --- |
| **Sepsis Resuscitation Bundle 6 hours, n (%)** | | | | |  |  | (n= 830) |
| Lactate measured | 964 (71.3) | 986 (77.3) | 635 (76.5) | 0.683 |  | | |
| Blood cultures before antibiotics | 656 (48.5) | 687 (53.8) | 492 (59.3) | 0.014 |  | | |
| Broad spectrum antibiotics | 903 (66.8) | 915 (71.7) | 587 (70.7) | 0.625 |  | | |
| Fluids and vasopressors | 815 (60.3) | 744 (58.3) | 476 (57.3) | 0.663 |  | | |
| **Time to antibiotics, mean (SD) hours^a^** | 2.5 (3.6) | 2.0 (2.7) | 2.2 (3.7) | 0.306 |  | | |
| **Evaluation of treatment at 72 hours, n (%)** 0.002 | | | | |  | | |
| Inappropriate treatment | 120 (8.9) | 83 (6.5) | 72 (8.7) |  |  | | |
| Appropriate treatment | 678 (50.1) | 654 (51.3) | 467 (56.3) |  |  | | |
| Negative cultures | 438 (32.4) | 417 (32.7) | 244 (29.4) |  |  | | |
| Cultures not done | 71 (5.3) | 56 (4.4) | 23 (2.8) |  |  | | |
| Patient died in less than 72 hours | 45 (3.3) | 66 (5.2) | 24 (2.9) |  |  | | |
| **Change of antibiotic at 72 hours, n (%)** 0.232 | | | | |  | | |
| Reduction of the spectrum | 220 (16.3) | 257 (20.1) | 181 (21.8) |  |  | | |
| No change of antibiotic | 762 (56.4) | 695 (54.5) | 437 (52.7) |  |  | | |
| Change due to poor clinical evolution | 143 (10.6) | 97 (7.6) | 76 (9.2) |  |  | | |
| Change due to uncovered microorganism | 82 (6.1) | 65 (5.1) | 50 (6) |  |  | | |
| Other | 145 (10.7) | 162 (12.7) | 86 (10.4) |  |  | | |

^a^Excluding patients with previous antibiotics (n = 761)
