# Supplementary material for: Improved empirical antibiotic treatment of sepsis after an educational intervention: the ABISS-Edusepsis study
Source: Crit Care. 2018 Jun 22;22:167. doi: 10.1186/s13054-018-2091-0 (PMC6013897; doi:10.1186/s13054-018-2091-0)
Supplement: Supplementary file 7 — Table S5. Outcome measurements in the long-term cohort. (DOC 31 kb) [file 13054_2018_2091_MOESM7_ESM.doc]

**Additional file 7: Table 5.** Outcome measurements in the long-term cohort

| **Outcome measurements** | **Preintervention cohort**  **(n=1352)** | **Postintervention cohort**  **(n= 1276)** | **Long-term cohort**  **(n= 830)** | **p** |
| --- | --- | --- | --- | --- |
| Duration of MV, days mean (SD) | 6.9 (14.4) | 6.6 (12.4) | 6.9 (11.3) | 0.623 |
| Duration of vasopressors, days mean (SD) | 4.0 (8.0) | 4.3 (7.0) | 4.6 (6.3) | 0.289 |
| ICU stay, days  mean (SD) | 12.0 (17.0) | 11.5 (14.9) | 11.5 (12.3) | 0.986 |
| Hospital stay, days mean (SD) | 30.0 (29.7) | 28.4 (28.9) | 27.2 (23.6) | 0.319 |
| **Mortality, n (%)** | | | | |
| ICU | 332 (24.6) | 301 (23.6) | 174 (21.0) | 0.159 |
| Hospital | 412 (30.5) | 375 (29.4) | 219 (26.4) | 0.134 |

Abbreviations: ICU, intensive care unit; MV, mechanical ventilation; SD, standard deviation.
