# Supplementary material for: Improved empirical antibiotic treatment of sepsis after an educational intervention: the ABISS-Edusepsis study
Source: Crit Care. 2018 Jun 22;22:167. doi: 10.1186/s13054-018-2091-0 (PMC6013897; doi:10.1186/s13054-018-2091-0)
Supplement: Supplementary file 8 — Table S6. Multivariate analysis of factors associated with mortality in the long-term cohort. (DOC 37 kb) [file 13054_2018_2091_MOESM8_ESM.doc]

**Additional file 8: Table 6.** Multivariate analysis of factors associated with mortality in the long-term cohort

| **Factors** | **OR** | **95% CI** | **P** |
| --- | --- | --- | --- |
| **Interventional cohort** | 0.83 | 0.67-1.03 | 0.091 |
| **Age^a^** | 1.02 | 0.64-1.01 | <0.001 |
| **Sex^b^** | 0.81 | 0.75-1.17 | 0.063 |
| **SOFA^a^** | 1.12 | 1.06-1.16 | <0.001 |
| **APACHE II^a^** | 1.07 | 1.05-1.09 | <0.001 |
| **CHARLSON^a^** | 1.09 | 1.04-1.14 | <0.001 |
| **Type of infection^c^** | | | |
| Nosocomial | 2.41 | 1.86-3.13 | <0.001 |
| ICU | 2.81 | 1.72-4.61 | <0.001 |
| Healthcare related | 1.12 | 0.83-1.69 | 0.351 |
| **Source of sepsis^d^** | | | |
| Acute abdominal infection | 0.74 | 0.57-0.96 | 0.023 |
| Urinary tract infection | 0.32 | 0.22-0.47 | <0.001 |
| Meningitis | 0.86 | 0.44-1.70 | 0.670 |
| Soft-tissue infection | 1.09 | 0.72-1.65 | 0.697 |
| Catheter-related bacteremia | 0.69 | 0.31-1.55 | 0.365 |
| Other infections | 0.91 | 0.56-1.50 | 0.716 |

Abbreviations: SOFA, Sequential Organ Failure Assessment; APACHE II, Acute Physiology and Chronic Health Evaluation II; ICU, Intensive Care Unit.

aPer each point of increase.

bCompared with male sex.

cCompared to community-acquired infection.

dCompared to pneumonia.
